# Repurposing nicardipine leads to improved development in a young patient with Pitt–Hopkins syndrome

**DOI:** 10.3389/fphar.2025.1592011

**Published:** 2025-07-25

**Authors:** Marta Agnes Somorai, Sean Ekins, Claudia Rupprecht, Clara Lettl, Volker Mall

**Affiliations:** ^1^ Center of Rare Diseases TUM – Development, Munich, Germany; ^2^ Kbo-Kinderzentrum Munich, Munich, Germany; ^3^ Technical University of Munich, TUM School of Medicine and Health, Chair of Social Pediatrics, München, Germany; ^4^ Collaborations Pharmaceuticals, Inc., Raleigh, NC, United States

**Keywords:** Pitt-Hopkins syndrome, precision treatment, nicardipine, TCF4 gene, drug repurposing

## Abstract

We describe a drug repurposing treatment involving the use of nicardipine in a young patient with Pitt-Hopkins syndrome (a rare neurodevelopmental disorder that results from variants of TCF4 gene) as a bench-to-bedside approach. Loss of TCF4 function in Pitt–Hopkins syndrome leads to increased excitability of Na_v_1.8 in neurons. Nicardipine is normally used alone or together with other medicines to treat severe chest pain (angina) or high blood pressure (hypertension), and can also be used in children to treat hypertension. Nicardipine was shown to have an inhibitory effect on Na_v_1.8 *in vitro* as well as in *Tcf4* ^*+/−*^ mice, showing promising effects on behavior, learning and memory. In this study, nicardipine was given orally for 7 months (starting dose 0.2 mg/kg/d, maximum dose 1.7 mg/kg/d). There were no significant side effects. The patient showed mild to moderate improvement in all developmental trajectories as well as in her restlessness. Repurposing nicardipine in Pitt-Hopkins syndrome patients could be a promising approach to enhance development in these often severely affected patients.

## Introduction

Pitt-Hopkins syndrome (PTHS, ORPHA:2896) is a well-known rare genetic disorder (Pitt and Hopkins, 1978) associated with heterozygous causative variants in the transcription factor 4 (*TCF4)* gene (Brockschmidt et al., 2007). Most patients show moderate to severe developmental delay/intellectual disability and behavioral problems, mostly from the autism spectrum, with severe involvement of speech development, breathing problems, hand stereotypes, distinctive facial features (broad mouth, widely spaced eyes), seizures and gastrointestinal problems. There is no cure in the form of precision therapy, and gene therapy for PTHS is still in development (Kim et al., 2022).

Dr. Brady Maher and colleagues studied cortical neurons from a PTHS mouse model (Rannals et al., 2016) and reported that TCF4 loss of function alters the intrinsic excitability of prefrontal neurons. While functional TCF4 repressed Na_v_1.8 (*SCN10A*), a voltage-gated ion channel in central neurons, loss of TCF4 function in PTHS led to increased Na_v_1.8 activity in the central nervous system (CNS). TCF4-dependent excitability was corrected via the use of small molecule Na_v_1.8 antagonists (Rannals et al., 2016). Therefore, targeting Na_v_1.8 with drugs could be a prospective therapeutic approach for PTHS to ameliorate cognitive and behavioral deficits.

We previously screened the Prestwick chemical library of FDA-approved drugs and drug-like molecules against stable clonal cell lines expressing Na_v_1.8 and identified 93 hits (7.2% hit rate) (Ekins et al., 2019). Nicardipine and nilvadipine were found to be the most potent inhibitors, with IC_50_ = 0.6 µM. Nicardipine was selected for *in vivo* testing because of its ability to cross the blood–brain barrier (Amenta et al., 2001) and impact on other CNS disorders (Flamm, 1989).

*Tcf4* ^*+/−*^ mice have been well characterized and have demonstrated deficits in habituation (Kennedy et al., 2016; T et al., 2018). A behavioral battery was used with *Tcf4* ^*+/−*^ mice to determine whether orally administered nicardipine (3 mg/kg once a day for 3 weeks) was sufficient to rescue hindlimb force control, learning and memory deficits, hyperactivity, sociability, nesting and stereotypy associated with PTHS (Ekins et al., 2020). Treatment of the mice with nicardipine did not have any side effects and led to a statistically significant improvement in all behavioral tests compared with vehicle-treated *Tcf4* ^*+/−*^mice, normalizing their behaviors to wild-type levels (Ekins et al., 2020).

Nicardipine is widely used as an FDA-approved therapy (calcium channel antagonist) available under prescription to treat angina and hypertension predominantly. It is also generally considered safe and is often used in children (Hosain et al., 1994; Lushaj et al., 2022). More recently, nicardipine has also been evaluated as a clinical treatment for neuroprotection of microglia (Huang et al., 2014) and has been shown to increase the clearance of beta amyloid (Bachmeier et al., 2011).

Previous studies have demonstrated CNS bioavailability (Sabbatini et al., 1995) when the drug is dosed orally or intravenously. The accessibility of nicardipine (20 mg and 30 mg doses (Anon)) and the fact that it has been used in millions of patients suggest the need for further assessment in PTHS, although it has not yet obtained FDA approval for use in pediatric populations.

Considering 1. the potential efficacy based on *in vitro* and *in vivo* animal studies (Ekins et al., 2020), 2. the well-known and moderate side effect profile of nicardipine, allowing thorough monitoring, and 3. the lack of approved therapies or promising clinical trials available for the family, we offered a therapeutic trial of nicardipine to our patient with PTHS. To our knowledge, this is the first report of repurposing nicardipine in a patient with PTHS.

## Case description

The patient presented to our outpatient clinic at the age of 2 years and 8 months with severe developmental delay, hand stereotypes, sleep disorders, muscle hypotonia, strabismus and typical facial dysmorphism (wide mouth, hypertelorism). She could turn to both sides, make sounds and concentrate 3–60 s on a task. Hand stereotypes could be observed in approximately 80% of her awake time, she could not grasp, but slap on pictures in a book held in front of her. EEGs were normal, and she had no seizures; therefore, an MRI of the brain was not performed. PTHS was diagnosed through trio-WES, a molecular genetic diagnostic approach analyzing all protein-coding genes of both parents and the affected child, revealing a pathogenic, *de novo*, heterozygous variant in the *TCF4* gene (c.656-1G>A, p.?). Chromosome analysis and Array-CGH were normal.

At the ages of 3 years and 2 months (=38 months), a standardized developmental survey (MFED, 90% – München Functional Developmental Survey) revealed developmental ages of 5 months for speech development, 6 months for fine motor development, 7 months for gross motor development and 7.5 months for perception (cognition), similar to the clinical impression 6 months earlier. She showed tremendous restlessness, making her mother busy 2–3 h/day trying to find a way to relieve her.

After the informed consent of the parents, approval through our ethics commission and normal results of a cardiology and basic laboratory workup, we initiated a therapeutic trial with nicardipine in our inpatient care system, allowing us to carefully monitor the clinical status, especially the blood pressure, of our patient. Nicardipine is not approved in Germany and was therefore imported from France. Nicardipine was introduced to the patient in a slow stepwise manner, with a starting dose of 2.5 mg at 7 a.m. (0.2 mg/kg/d), followed by 2.5 mg at 7 a.m. and at 2 p.m., 7 days later. 0.5 mg dose increases every 7 days in the first 3 weeks and every 3–4 days thereafter followed, up to a maximum dose of 10 mg twice a day (1.7 mg/kg/d), which we reached 3 months later. Nicardipine was given for 4 months at the maximum dose, followed by stepwise (2.5 mg steps every 3 days) withdrawal.

**FIGURE 1:**
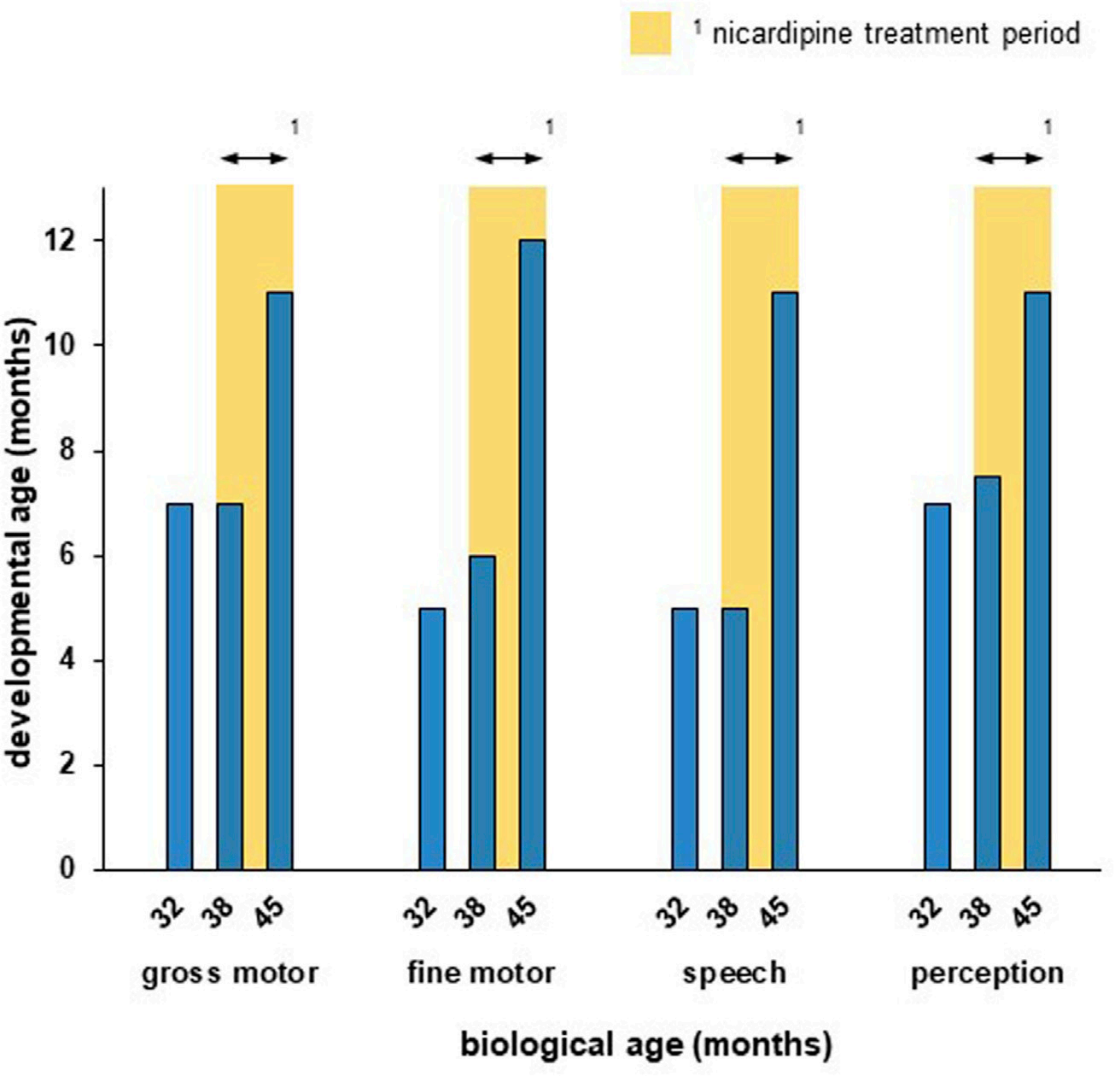
The patient showed measurable improvement in all developmental trajectories during the nicardipine treatment period. Developmental age was assesed according to the standardized developmental survey (MFED, 90%). The nicardipine treatment period is indicated through yellow bars. While the developmental progress was little between the first (32 months) and second (38 months) survey, a strong improvement in all developmental trajectories could be observed after 7 months of nicardipine treatment (45 months).

The girl showed improvement in all developmental trajectories under nicardipine (Figure 1). Although she reached only the developmental milestone turning to both sides at the age of 3 years, she learned to come onto all fours on her own during titration and learned to walk held on a finger on each hand during the 4 months of treatment. She showed mild improvement in hand motor function, as she learned to turn a page in a cardboard book. During treatment, she started making syllables and using one first word with meaning (mama) as well as first signs (no and give). She was calmer and needed less support and calming from her mother (ca. 1 h/day), she could concentrate longer on a task (2–3 min). At the biological ages of 3 years and 9 months, i.e., at the end of the treatment trial, the developmental survey (MFED, 90%) revealed developmental ages of 11 months for gross motor, speech development and perception (cognition), and 12 months for fine motor development (Figure 1). There were no significant changes in sleep.

Nicardipine had a slight effect on blood pressure, especially at the beginning of the trial (baseline: 101–111/56–67; within 6 h after nicardipine: 86–99/50–63). Her systolic blood pressure was above 85 mmHg at all times. The patient did not seem sedated, and there was no need for medical intervention at any time. The only side effect was reddening of the cheeks for 30 min - 2 h after the morning dose, especially after dose escalation. No gastrointestinal side effects or changes in pain-sensitivity were reported.

No objective developmental regression was observed following therapy withdrawal. The family decided to reintroduce the nicardipine treatment after a treatment pause to further enhance the patient’s development, since especially her speech production and overall communicative competence were gradually decreasing in the year off nicardipine. As of submission, preparations are made for the planned reintroduction (cardiology clearance, import of nicardipine).

## Discussion and conclusion

The rationale for offering a drug repurposing approach for pediatric patients with chronic and debilitating genetic disorders is based on a wide spectrum of evidence, from rarely available randomized controlled clinical trials over case series and animal models up to expert opinions including a thorough understanding of the cellular pathomechanism and mechanism of action of the suggested drug. In this case, *in vitro* and *in vivo* experimental models showed a gain of function of the neuronal Nav 1.8 ion channel secondary to the loss of function of TCF4 in Pitt-Hopkins syndrome.

Monogenic disorders of single ion-channels (channelopathies) represent a significant portion of monogenic neurodevelopmental disorders. Classic symptoms of these disorders being developmental delay and/or epilepsy. Available precision therapy approaches not only provide benefits in the treatment of epilepsy, but also in the improvement of the development, even without an epilepsy (Gjerulfsen et al., 2024). Therefore, a treatment approach for a neurodevelopmental disorder directed to restore a disturbed ion-channel function seemed promising and worthy of further investigation.

The preclinical studies involved drug-screening resulting in the non-selective Nav 1.8 inhibitor nicardipine becoming the selected drug for the consequent studies in the TCF4 knock out mouse model. *In vivo* animal models for genetic neurodevelopmental disorders commonly involve mouse models to narrow drug candidates prior to human administration, although mouse as an *in vivo* model, especially young mice, underestimate the complexity of the human brain structure and function. Examples of this approach include the case of trofinetide (Daybue^®^), a medication approved in March 2023 through the FDA for the treatment of Rett syndrome. Tropea and colleagues had published in 2009 the *in vivo* data in mouse model leading to clinical trials in humans (Tropea et al., 2009).

The treatment of a young girl with PTHS using nicardipine over a total period of 7 months resulted in developmental progress above expectations in all developmental trajectories without relevant side effects. We assume that off-label treatment with nicardipine temporarily increased the patient´s developmental velocity. Additionally, it speaks for the positive effect of the treatment, that the family decided to reintroduce the nicardipine treatment after a treatment pause, despite the frequent blood pressure controls the therapy requires.

A larger clinical trial is certainly necessary to clearly show the effect of repurposing nicardipine in PTHS children. We would suggest the implementation of a two-way crossover design to consider the normal development of patients (under placebo) as well. Furthermore, other tests than those used in this study should be explored to capture the broad effects of the disease on motor ability, intellectual ability, breathing, seizures and quality of life. A larger clinical trial would also require significant funding and may need to be a multicenter study, as this is a rare disease with very few patients in each country.

Other Na_v_1.8 inhibitors, that are more specific to this sodium channel could be considered in the future for treating this rare disease, if they are able to cross the blood-brain barrier and consequently CNS activity can be demonstrated. This would exclude the recently FDA approved VX-548, which is used for treating pain but lacks CNS activity (Jones et al., 2023).

## Data Availability

The original contributions presented in the study are included in the article/supplementary material, further inquiries can be directed to the corresponding author.

## Abbreviations

PTHS, Pitt–Hopkins syndrome; TCF4, transcription factor 4; SCN10a, Na_v_1.8 ion channel; FDA, U. S. Food and Drug Administration; CNS, central nervous system; WES, Whole-Exome Sequencing; Array-CGH, Array-Comparative Genomic Hybridization; EEG, electroencephalogram; MRI, magnetic resonance imaging; MFED, München Functional Developmental Survey.
